# The effects of hippocampal lesions on MRI measures of structural and functional connectivity

**DOI:** 10.1002/hipo.22621

**Published:** 2016-08-24

**Authors:** Richard N. Henson, Andrea Greve, Elisa Cooper, Mariella Gregori, Jon S. Simons, Linda Geerligs, Sharon Erzinçlioğlu, Narinder Kapur, Georgina Browne

**Affiliations:** ^1^ MRC Cognition & Brain Sciences UnitCambridgeEngland; ^2^ Neuropsychology DepartmentAddenbrooke's Hospital, Cambridge University Hospitals NHS Foundation TrustCambridgeEngland; ^3^ Department of PsychologyUniversity of CambridgeCambridgeUnited Kingdom; ^4^ Research Department of Clinical, Educational and Health PsychologyUniversity College LondonLondonUnited Kingdom

**Keywords:** hippocampus, amnesia, diaschisis, functional connectivity, structural connectivity, VBM, DTI

## Abstract

Focal lesions can affect connectivity between distal brain regions (connectional diaschisis) and impact the graph‐theoretic properties of major brain networks (connectomic diaschisis). Given its unique anatomy and diverse range of functions, the hippocampus has been claimed to be a critical “hub” in brain networks. We investigated the effects of hippocampal lesions on structural and functional connectivity in six patients with amnesia, using a range of magnetic resonance imaging (MRI) analyses. Neuropsychological assessment revealed marked episodic memory impairment and generally intact performance across other cognitive domains. The hippocampus was the only brain structure exhibiting reduced grey‐matter volume that was consistent across patients, and the fornix was the only major white‐matter tract to show altered structural connectivity according to both diffusion metrics. Nonetheless, functional MRI revealed both increases and decreases in functional connectivity. Analysis at the level of regions within the default‐mode network revealed reduced functional connectivity, including between nonhippocampal regions (connectional diaschisis). Analysis at the level of functional networks revealed reduced connectivity between thalamic and precuneus networks, but increased connectivity between the default‐mode network and frontal executive network. The overall functional connectome showed evidence of increased functional segregation in patients (connectomic diaschisis). Together, these results point to dynamic reorganization following hippocampal lesions, with both decreased and increased functional connectivity involving limbic‐diencephalic structures and larger‐scale networks. © 2016 The Authors Hippocampus Published by Wiley Periodicals, Inc.

## INTRODUCTION

The hippocampus is probably one of the most scrutinized regions of the brain, given its unique neural architecture (Anderson et al., 2007) and diverse range of functions (e.g., Eichenbaum and Cohen, 2014; Strange et al., 2014). Ever since documentation of the profound amnesia caused by hippocampal resection (Scoville and Milner, 1957), the hippocampus has been studied in both animals and humans, and implicated not only in memory (e.g., Squire, 1992), but also in spatial maps (e.g., O'Keefe and Nadel, 1979), emotional processing (e.g., Kim and Fanselow, 1992), relational processing (e.g., Cohen and Eichenbaum, 1994), scene construction (e.g., Zeidman et al., 2015), and perception (e.g., Graham et al., 2010). One reason for its considerable behavioral significance is likely to be its central role in interactions with other brain regions, i.e., in terms of its structural and functional connectivity (Aggleton and Brown, 1999). Here we used T1‐weighted, diffusion‐weighted and blood‐oxygenation‐level‐dependent (BOLD)‐weighted magnetic resonance imaging (MRI) on a group of 6 patients with hippocampal lesions, to examine the “knock‐on” effects of these lesions on structural and functional connectivity.

Awareness of the implications of focal lesions for brain connectivity has recently been revitalised in terms of “connectional diaschisis”—changes that occur to brain regions distant from the location of the lesion (Carrera and Tononi, 2014). While connections to and from lesioned regions are likely to suffer owing to lost afferents (e.g., Campo et al., 2012), connectional diaschisis can also involve connectivity changes between distal regions that are not directly connected to the lesion site. Furthermore, one can examine the effect of lesions on the properties of a larger network, such as its functional segregation. This form of “connectomic diaschisis” (Carrera and Tononi, 2014) can reveal, for example, whether a lesion has affected a “provincial hub” or “connector hub” (Sporns et al., 2007). Hubs are brain regions with relatively high numbers of connections, of which provincial hubs link mainly regions within the same subnetwork (module), whereas connector hubs link many different subnetworks. More generally, the study of connectomics is interesting from a clinical as well as theoretical perspective, in providing insights into both adaptive and maladaptive processes in brain disorders (Fornito et al., 2015).

A few studies have used tensor analysis of diffusion‐weighted MR images (diffusion tensor imaging, DTI) to examine effects of hippocampal lesions on structural connectivity, i.e., white‐matter integrity (WMI). At least two studies have examined diffusion properties in nonhuman primates (NHP) following neurotoxic lesions in the hippocampus (Meng et al., 2014; Shamy et al., 2010) and found evidence of reduced WMI in the fornix, which carries the major input and output of the hippocampus. Nonetheless, evidence for damage to other major white‐matter tracts in these NHP studies is mixed (see Discussion). Some studies have used DTI in groups of human patients with mesial temporal lobe epilepsy (e.g., Liao et al., 2011; Voets et al., 2009), and found evidence of reduced WMI between the hippocampus and posterior cingulate. However, epilepsy often leads to damage that extends beyond the hippocampus (Spencer, 2002), which leaves open the question of whether effects are specific to the hippocampus. One human MRI study examined the effect of acquired, adult‐onset hippocampal lesions (Rudebeck et al., 2013). This study examined two patients, and found reduced fractional anisotrophy (FA) of the fornix. However, these reductions only reached significance in the patient with the larger medial temporal lobe (MTL) lesions, encompassing, for example, parahippocampal regions as well.

Other MRI studies have investigated the effect of hippocampal lesions on functional rather than structural connectivity, using BOLD‐weighted fMRI. Some of these examined the impact of neurodegenerative processes, such as Alzheimer's disease (e.g., Buckner et al., 2009; Greicius et al., 2004), but Alzheimer's disease pathology can extend outside the hippocampus. Other studies have used patients with mesial temporal lobe epilepsy (e.g., Liao et al., 2011; Voets et al., 2009), but, as well as the likelihood of extra‐hippocampal damage, the effect of the patient's medication on the fMRI BOLD signal remains unclear. We are only aware of two studies using patients with focal, adult‐onset hippocampal lesions. The patient with the larger MTL lesion in the aforementioned Rudebeck et al. (2013) study showed reduced connectivity within a “posterior hippocampal network” during rest. A second study, by Hayes et al. (2012), examined three cases of amnesia with MTL damage. These authors chose a seed in posterior cingulate cortex (PCC) to explore functional connectivity with other areas in the “default mode network” (DMN; Greicius et al., 2004) that includes the MTL. Perhaps unsurprisingly, given the tissue loss in MTL, they found reduced functional connectivity between the PCC seed and MTL voxels. However, they also found increased connectivity between the PCC seed and voxels in other DMN regions, such as medial prefrontal cortex, retrosplenial cortex and posterior inferior parietal regions. This could reflect some type of “disinhibition” of the DMN owing to MTL lesions. However, the relatively large size of the lesions in these patients again precludes confident attribution of any such connectional diaschisis to the hippocampus. Moreover, these studies did not examine the effect of hippocampal lesions on the other large functional networks known to exist in the brain (e.g., Geerligs et al., 2015), nor on the graph properties of global brain organization (i.e., connectomic diaschisis).

This study examined a human sample of six amnesic individuals with focal, adult‐acquired hippocampal lesions, and applied a range of MRI measures and analyses to investigate the extent of hippocampal damage, and its impact on structural and functional connectivity with, and outside, the hippocampus, as well as on large‐scale networks and whole‐brain functional connectomics. The results clearly demonstrate the central role of the hippocampus in the brain's major functional networks.

## MATERIALS AND METHODS

### Participants

The 6 patients were selected from the Cambridge Hippocampal Panel, as approved by NRES Ethics Committee East of England (ref 12/EE/0190) and consent obtained according to the Declaration of Helsinki, on the basis of reported memory difficulties and, in some cases, a diagnostic MR scan that showed an indication of limited MTL damage, with various etiologies (Table 1). Case histories and previous cognitive assessments are described below; the new neuropsychological data acquired for the present study are reported in the Results section.

**Table 1:** Patient Details

PATIENT	P1	P2	P3	P4	P5	P6
Age (years)	57	39	66	66	57	62
Gender	male	male	male	male	female	male
Education (years)	Left school prior to O‐levels	14	12+ Apprenticeship	14+	14+	12+ Apprenticeship
Presenting diagnosis	Complex hypoxia	Carbon monoxide poisoning	Carbon monoxide poisoning	Limbic encephalitis	Limbic encephalitis	Limbic encephalitis
History of symptoms (years)	unknown, 1	20	18	6	6	14, 4

See text for further details, e.g., history of symptoms.

#### P1

P1 was a 57‐year‐old right‐handed male, who presented with marked retrograde and anterograde memory impairments following an episode of hypoxia with seizure activity, 1 yr prior to testing. For an unknown period prior to this episode, there is some suspicion that he had limbic encephalitis, following an MRI consistent with this diagnosis. He also had a history of respiratory illnesses, including lung cancer, and a period of moderate alcohol misuse, though neither he nor his family reported any memory problems during this period, and he was able to hold down jobs. He left education prior to completing O‐Levels. He showed marked memory deficits, including qualitatively poor autobiographical memory, and mild symptoms of depression (though it is unlikely that depression was contributing significantly to his densely amnestic profile). Thus, while we think that his amnesia was secondary to the hypoxic episode, which was secondary to a seizure, we cannot be sure of the precise etiology. He was not oriented to the date and year. Other domains of cognitive functioning remained intact. His estimated level of pre‐morbid cognitive functioning, as suggested by adult reading test score, was in the normal range. A diagnostic MRI reported hyperintensity of MTL structures.

#### P2

P2 was a 39‐year‐old right‐handed male artist and carer for his young son, who presented with a 20‐year history of severe amnesia following carbon monoxide poisoning and subsequent seizure activity. He left education with 3 A‐levels, and, postbrain injury, he completed a film studies course. Previous tests showed marked impairment on tests of episodic memory. His autobiographical memory remained relatively intact, with the exception of details from near the time of injury. With effort and a complex system of reminders and strategies, he was able to accomplish daily tasks and to orient to the date and year. There was no indication of impairments in other domains of cognitive functioning, or of depression or anxiety. Premorbid cognitive functioning as assessed by an adult reading test was above average.

#### P3

P3 was a 66‐year‐old right‐handed male, who presented with a history of anterograde and retrograde amnesia subsequent to carbon monoxide poisoning 18 years prior to testing. After leaving school at 16, he completed an apprenticeship and prior to injury worked as an insurance salesman. His memory for autobiographical experiences and details for remote and current events was impaired. He was not oriented to date, time, or location. There were no other impairments of note, and there was no indication of depression or anxiety. Performance was above average on an adult reading test. An initial diagnostic MRI scan showed globus pallidus lesions and hippocampal volume loss, and an initial positron emission tomography (PET) scan showed bilateral MTL hypometabolism, slightly more so on the right. (Note however that formal comparison of grey‐matter volume (GMV) did not reveal significant loss in pallidum; see Results).

#### P4

P4 was a 66‐year‐old right‐handed male, who had a history of memory impairment following an episode of limbic encephalitis 6 years prior to testing. Tests for autoimmune disease were negative, suggesting an infectious cause (e.g., herpes simplex). He studied zoology at university, and prior to his illness worked as a lecturer in that field. He demonstrated some confabulation along with poor memory for autobiographical details and current events. He was not oriented to date. With extensive rehearsal and memory aids he was able to manage some simple daily routines. There was evidence of flat affect and some impairment in other domains of cognitive function. Performance on a test of adult reading was in the high average range. An initial diagnostic MRI scan showed GMV loss in the right anterior hippocampus.

#### P5

Patient P5 was a 57‐year‐old right‐handed female, who presented with a 6‐year history of memory difficulties following limbic encephalitis and subsequent seizure activity. The etiology of the encephalitis is unknown. She studied mathematics at university and was a qualified teacher before working as a civil servant. She later worked in the family business, where, post‐illness, she continued with some general office duties and proof reading. It is likely that her high premorbid ability, supportive environment and compensatory strategies improved her performance on formal neuropsychological tests. Nonetheless, she had difficulty orienting to new environments, and demonstrated poor autobiographical memory for events in the previous 6 years (e.g., could not recall significant recent public events and was unable to recall events from the week preceding her neuropsychological appointment). She was oriented in time, and there were no additional cognitive impairments of note. A diagnostic MRI scan showed bilateral hippocampal volume loss.

#### P6

P6 was a 62‐year‐old male, who presented with memory difficulties following two episodes of limbic encephalitis from herpes simplex, 14 and 4 years prior to testing. After completing his O‐level qualifications and an apprenticeship, he worked as a qualified car mechanic until the second episode of encephalitis. He was easily disoriented, and suffered from mild anxiety and depression, though his language skills, auditory attention, and general functioning were relatively preserved. A diagnostic MRI scan established cerebral damage and shrinkage in bilateral temporal regions.

#### Controls

Matched control participants were members of the Medical Research Council, Cognition and Brain Sciences Unit's (MRC CBU) MRI database of several thousand healthy volunteers who had been scanned at the CBU over the last 5 years. The specific details for each control group varied per analysis and are provided in the Results section.

### Neuropsychological Assessment

All patients undertook a battery of standardized neuropsychological tests to determine their current level of functioning across five cognitive domains: memory (verbal and visuospatial); verbal skills; visuospatial skills, attention, and executive functioning. Memory was measured using tests from the BIRT (Brain Injury Rehabilitation Trust) Memory and Information Processing Battery (BMIPB; Coughlan et al., 2007) and the Warrington recognition memory test (RMT) (Warrington, 1984). All patients also completed the National Adult Reading Test (NART) to help estimate level of premorbid cognitive functioning (Nelson and Wilson, 1991) and the self‐reported Hospital Anxiety and Depression Scale (HADS) (Zigmond and Snaith, 1983). For P1–P4, the Similarities, Vocabulary, Block Design, Matrix reasoning, Digit Symbol, and Digit Span tests were from the Wechsler Adult Intelligence Scale, 3rd edition (WAIS III) (Wechsler, 1997); for P5 and P6, the Digit Symbol and Digit Span tests were from WAIS IV (Wechsler, 2008), and the remaining tests were from the Wechsler Abbreviated Scale of Intelligence (WASI; Wechsler, 1999).

### MRI Data

The T1‐weighted structural images, diffusion‐weighted images (DWIs) and BOLD‐weighted (functional) MRI images were acquired using a 12‐channel headcoil on a Siemens 3 T TIM Trio system at the MRC CBU, during a 30 min procedure.

### Structural MRIs

The high‐resolution, 3D T1‐weighted images were acquired using a Magnetisation Prepared RApid Gradient Echo (MPRAGE) sequence with the following parameters: repetition time (TR) = 2250 ms; echo time (TE) = 2.98 ms; inversion time (TI) = 900 ms; flip angle = 9°; field of view (FOV) = 256 × 240 × 192 mm; voxel size = 1 mm isotropic; GRAPPA acceleration factor = 2; acquisition time = 4.5 min.

The DWIs were acquired using a 2D echo‐planar imaging (EPI), twice‐refocused spin‐echo sequence, with 64 diffusion gradient directions with *b* = 1000 s/mm^2^ plus one non‐weighted image with *b* = 0 s/mm^2^, TR = 8400 ms, TE = 90 ms, voxel size = 2 mm isotropic, FOV = 192 × 192 mm, 68 axial slices, GRAPPA acceleration factor = 2; acquisition time = 9.5 min.

The BOLD‐weighted fMRI data were acquired using a Gradient‐Echo EPI sequence. Participants were told to lie still, close their eyes and relax, not to think about anything in particular, and not to fall asleep. A total of 150 volumes are acquired, each containing 32 axial slices (acquired in descending order), with slice thickness of 3.75 mm and an interslice gap of 20% (to cover most of the brain, except the inferior most portion of the cerebellum); TR = 2000 ms; TE = 30 ms; flip angle = 78°; FOV = 192 × 192 mm; voxel‐size = 3 × 3 × 4.5 mm) and acquisition time of 5 min. The first two volumes were discarded for T1 equilibration.

#### Subcortical volume

The T1‐weighted images were segmented using FreeSurfer (FS) version 5.0.7 (http://surfer.nmr.mgh.harvard.edu/). We used the automated reconstruction protocol “recon‐all” which has been described previously (Fischl et al., 2004). Briefly, this pipeline includes motion correction, removal of nonbrain tissues, affine registration into Talairach space and segmentation of the subcortical white‐ and grey‐matter structures. This automated segmentation procedure assigns anatomical labels to each voxel using probabilistic information derived from a manually labeled training set, which constrains the location of structures in relation to each other. The accuracy of this segmentation is illustrated for each patient in Supporting Information, Figure 5. Regional volumes were extracted for subcortical regions of interest (ROIs).

#### Voxel‐based morphometry

Voxel‐wise, whole‐brain analysis of the T1‐weighted images was performed in SPM8 (http://www.fil.ion.ucl.ac.uk/spm), together with Automatic Analysis Version 4 (https://github.com/rhodricusack/automaticanalysis). This included an initial step of bias correction for field inhomogeneity, followed by unified segmentation and normalization (“Seg8”) (Ashburner 2007). The resulting grey‐matter images for each participant were coregistered to their average using an iterative, diffeomorphic method called DARTEL (Ashburner, 2007). Note that each group (consisting of a patient and their matched controls) was registered separately. The resulting images were then affine normalized to Montreal Neurological Institute (MNI) space. The grey‐matter density in each voxel was modulated by the local warping entailed by coregistration and normalization, to produce an estimate of local GMV. These estimates were smoothed by an 8 mm isotropic Gaussian kernel, and scaled by total grey‐matter in the native grey‐matter image. For small‐volume correction, the hippocampus was defined by the maximal probability anatomical LPBA40 LONI atlas (http://www.loni.usc.edu/atlases/Atlas_Detail.php?atlas_id = 12; Shattuck et al., 2007).

#### Diffusion tensor imaging

MRIcron (http://www.mccauslandcenter.sc.edu/mricro/mricron/) was used to convert the DICOM files to NIFTI images, including the files of magnetic field (B0) values and vectors. Correction for eddy currents and simple head motion was performed using the FDT module of the FSL Software Library (FMRIB Software Library 5.0.6). Eddy current and motion‐corrected files were used for Tract‐Based Spatial Statistics (TBSS) (Smith et al., 2006). For TBSS analysis, FA images were created by fitting a tensor model to the raw diffusion data using the FDT module in FSL, followed by brain‐extraction using the BET module (Smith, 2002). All participants' FA data were aligned to the MNI 152 atlas using the nonlinear registration tool FNIRT. The transformed maps were averaged to generate a mean FA image, which was ‘thinned’ using an FA threshold of 0.2 to create a white‐matter skeleton, representing the centers of all FA tracts common to the group. FSL's “randomize” function with 500 permutations was used to produce T‐statistics for each voxel in the skeleton, and corrected for multiple comparisons using false discovery rate (FDR) of 0.05.

Given that there were more controls (36) than patients (6), two approaches were taken to defining the white‐matter skeleton: (i) a “matched skeleton” averaged across the 6 patients and a randomly selected subset of 6 controls, and (ii) a “complete skeleton” averaged across all 42 controls and patients. Similar results with both sets of skeletons would ensure that our findings were not influenced by the unbalanced number of controls and patients used to define the skeleton (Bach et al., 2014). Aligned FA images of all controls and patients were projected onto each of the two FA skeletons, and then the mean FA calculated across all voxels within both the skeleton and each of four white matter ROIs. We also repeated this for mean diffusivity (MD; 10^−3^ mm^2^ s^−1^), given that it may provide complementary information on white‐matter microstructure (Beaulieu, 2002). These ROIs were the main tracts known to connect to MTL (Catani et al., 2008), as defined by the Johns Hopkins University (JHU) DTI‐81 atlas (http://cmrm.med.jhmi.edu/): (1) Fornix (body), (2) Hippocampal Cingulum, (3) Uncinate Fasciculus, and (4) Inferior Longitudinal Fasciculus.

### Functional MRI

#### fMRI preprocessing

Preprocessing was performed using the SPM12 software (http://www.fil.ion.ucl.ac.uk/spm). The functional images were motion‐corrected and slice‐time corrected, coregistered to the T1 image, and normalized to MNI space.

FMRI functional connectivity analysis is notoriously sensitive to motion artifacts. The motion parameters (3 translations and 3 rotations) were estimated from spatial realignment, and, together with the mean aligned image, converted into a single root mean square relative displacement (RD) using the approach of Jenkinson et al. (2002). Two of the controls and one patient (P6) were outliers in terms of their mean RD, relative to the interquartile range of the controls (Supporting Information, Fig. 1). Analysis was therefore repeated with and without these three individuals.

To reduce the effects of motion on the functional connectivity results, we used a combination of approaches. The first of these was to apply the Wavelet Despike method for removing motion artifacts from fMRI data without the need for data scrubbing (Patel et al., 2014). The method detects irregular events at different frequencies by identifying chains of outlying wavelet coefficients, and removes these from voxel time series. The algorithm can remove both prolonged motion artifacts, such as spin‐history effects, as well as higher frequency events such as spikes. The second and third methods to address motion are described below, after the data were reduced to ROIs.

#### ROI definitions

Images of despiked data for each voxel were then reduced to a smaller number of ROIs. The *N* = 8 functional ROIs previously shown to be connected to the hippocampus were taken from the dorsal DMN defined by http://findlab.stanford.edu/functional_ROIs.html (Shirer et al., 2011), though a small right prefrontal cluster (labeled 5) was merged with its homologous left cluster in the larger medial prefrontal ROI (labeled 3). For the whole‐brain network and connectomic analyses, 748 functional ROIs were taken from Geerligs et al. (2015), which were in turn based on the functional parcellation from a previous study (Craddock et al., 2012). Because the present data did not cover the most inferior part of the cerebellum in all participants, 32 of these ROIs were excluded because fewer than 50% of their voxels overlapped with the present fMRI data in one or more participants (leaving 716 ROIs common to all participants).

We also added a “lesion” ROI reflecting voxels in both left and right hippocampal clusters that showed significantly reduced GMV for the patient group relative to control group, plus three “artifact” regions defined by voxels with a 75% or more probability of being 1) WM or 2) cerebrospinal fluid (CSF), plus 3) all voxels within the intracranial volume. The timeseries for each region was the first temporal mode of a singular‐value decomposition (SVD) across voxels in that ROI (to capture the “dominant” temporal pattern, downweighting atypical voxels). The sign was adjusted to match that of the mean across voxels, and standardized (Z‐scored), to have unit variance over volumes.

#### Functional connectivity estimates

Functional connectivity was estimated using a general linear model (GLM) in which the timeseries in one (target) ROI was regressed against the timeseries in another (seed), together with additional regressors to capture confounding effects of no interest (and this repeated for all *N×*(*N*−1) unique pairs of the *N* ROIs). These confounds included the timeseries from the 3 “artifact” regions, plus a second‐order, lag‐five Volterra expansion of the RD index of motion. This Volterra expansion represents the second method used to control for motion, by allowing for linear, quadratic (Satterthwaite et al., 2013) and delayed (up to 5 TRs, Power et al., 2014) effects of movement, e.g., due to movement‐by‐distortion interactions and spin‐history effects, with basic functions covering both five individual TRs and the 4 successive differences between these TRs (producing 90 regressors in total). These movement regressors, together with the 3 confound timeseries, were subjected to a SVD, given their high intercorrelation, and only those components maintained that were needed to explain at least 99% of variance (13 on average). Finally, a discrete cosine set of 94 components was added to the regression model, to implement a band‐pass filter from 0.009–0.1 Hz, further removing nonhemodynamic noise sources. Inclusion of all these covariates left 41 residual degrees of freedom (dfs) on average.

The autocorrelation in the GLM error was modeled by a family of 8 exponentials with half‐lives from 0.5 to 64 TRs, given evidence that an AR(1) plus white noise model is not sufficient for resting‐state connectivity (Eklund et al., 2012). The GLM parameters and autocorrelation hyperparameters were estimated simultaneously by Restricted Maximum Likelihood Estimation (ReML) (Friston et al., 2002). The result of this GLM is a *Z*‐statistic that correctly accounts for the dfs lost by removing confounds, filtering, and temporal autocorrelation in the error. The Matlab code for this procedure can be found here http://www.mrc-cbu.cam.ac.uk/people/rik.henson/personal/analysis. For each connection, *Z*‐statistics were averaged across the two cases with each ROI as the seed (resulting in symmetric connectivity matrices).

The third and final correction for motion was to regress out from each connection the RD motion estimate across all participants (controls and patients combined; Yan et al., 2013).

### Statistics

Where separate groups of age‐ and sex‐matched controls were available (see Results), patients were tested against their own controls using a *T*‐test with pooled variance (equivalent to Crawford and Howell's (1998) *T*‐test; http://www.mrc-cbu.cam.ac.uk/personal/rik.henson/personal/Henson_Singlecase_06.pdf); where only a single group of controls was available, a single unpaired *T*‐test was used to compare patient and control groups. *P* values are two‐tailed.

For the graph‐theoretic measures, we estimated global clustering and global efficiency for undirected, binarized connections between the 716 Craddock ROIs using the Brain Connectivity Toolbox (https://sites.google.com/site/bctnet/; Rubinov and Sporns, 2010). We tested percentile thresholds from *P* = 85% to 99% (in steps of 1%): i.e., maintaining only those connections with the top *P*% of *Z* values (this matches the number of connections, or network degree, across participants). This binarization allows us to focus on the pattern of connections, rather than overall differences in connectivity strength. Global clustering was the mean clustering coefficient across nodes (ROIs), where the clustering coefficient is the fraction of neighbors of a node that are also neighbors of each other. Global efficiency is the average of inverse shortest path length, and inversely related to the characteristic path length. Small‐worldness is the ratio of clustering to characteristic path length, so was approximated as the product of global clustering and global efficiency.

Given that high binarization thresholds can result in “isolated” nodes (that are not connected to at least one other node), which obscure interpretation of some graph‐theoretic measures, we calculated the proportion of such isolates at the thresholds for which group differences in small‐worldness and clustering were most significant (97% and 99%, respectively; see Results). At the 97% threshold, the median proportion of such isolates was 1.33% for patients and 0.559% for controls, which did not differ significantly according to a nonparametric rank sum test (*P* = 0.742). At the 99% threshold, the median proportion of such isolates was 12.5% for patients and 13.4% controls, which did not differ significantly according to a nonparametric rank sum test (*P* = 0.858). These data suggest that the group differences in graph‐theoretic properties did not owe to differences in the proportion of isolated nodes.

## RESULTS

### Neuropsychological Assessment

Standardized neuropsychological scores were compared to normative data, matched for age and education where available (Table 2). In terms of premorbid ability, all but one patient (P1) had higher than average IQ, as estimated from the NART. Only P1 and P6 reported mild anxiety and/or depression.

**Table 2:** Neuropsychological Tests for the 6 Patients

	Neuropsychological tests					
Patient	P1	P2	P3	P4	P5	P6
Verbal memory						
BMIPB Story Recall – Immediate (60)	15	5***	9***	6***	21*	21*
	5th–10th %ile	<2nd %ile	<2nd %ile	<2nd %ile	10th–25th %ile	5th–10th %ile
BMIPB Story Recall – Delayed (60)	3***	0***	0***	0***	14*	10***
	<2nd %ile	<2nd %ile	<2nd %ile	<2nd %ile	10th–25th %ile	< 2nd %ile
Recognition Memory Test—Words (50)	32***	47	42	10 (25)***	47	35*
	<5th %ile	50th %ile	50th %ile	discontinued	75th–95th %ile	5th %ile
Visuospatial Memory						
BMIPB complex figure – Immediate (80)	16***	38***	13***	9***	41**	49
	<2nd %ile	<2nd %ile	<2nd %ile	<2nd %ile	2nd–5th %ile	25th–50th %ile
BMIPB complex figure – 40 min (80)	0***	13***	0	0***	41*	32
	<2nd %ile	<2nd %ile	<2nd %ile	<2nd %ile	10th–25th %ile	10th–25th %ile
Recognition Memory Test—Faces (50)	24***	42	44	29**	42	40
	<5th %ile	25th–50th %ile	50th–75th %ile	<5th %ile	50th %ile	25th–50th %ile
Verbal Skills						
NART predicted premorbid ‘IQ’	91	112	123	118	121	111
					
Graded Naming Test (30)	25	25	22	27	21	22
	14th %ile	14th %ile	50th–75th %ile	95th–99th %ile	50th–75th %ile	50th–75th %ile
Letter Fluency – FAS	31	34	32	51	36	41
	10th–20th %ile	20th–30th %ile	40th %ile	70th–80th %ile	20th–30th %ile	50th %ile
Category Fluency – Animal	15	17	12*	16*	19	19
	10th%ile	10th–25th %ile	10th %ile	25th %ile	25th–50th %ile	50th %ile
WAIS/WASI Similarities	21 (33)	31 (33)	31 (33)	28 (33)	42 (48)	41 (48)
	SS 10	SS 15	SS 17	SS 14	TS 63	TS 61
WAIS/WASI Vocabulary	46 (66)	RSNA	62 (66)	TNA	75 (80)	71 (80)
	SS 11	SS 14	SS 16		TS 68	TS 64
Visuospatial skills						
BMIPB complex figure—Copy (80)	75	80	80	80	79	RSNA
	10th–25th %ile	≥ 75th %ile	≥ 75th %ile	≥ 75th %ile	≥ 75th %ile	≥ 75th %ile
WAIS/WASI Block Design	22 (68)	60 (68)	46 (68)	10 (68)***	44 (71)	41 (71)
	SS 7	SS 16	SS 14	SS 4	TS 57	TS 55
WAIS/WASI Matrix Reasoning	19 (26)	24 (26)	21 (26)	9(26)	27 (32)	24 (32)
	SS 13	SS 16	SS 15	SS 8	TS 62	TS 58
Sustained Attention						
WAIS Digit Symbol	48	87	45	9***	74	56
	SS 7	SS 12	SS 8	SS 2	SS 13	SS 9
WAIS Digit Span—Forward	TNA	6	7	5	5*	7
		SS 11	SS 11	SS 7	SS 8	SS 10
WAIS Digit Span—Backward	TNA	6	4	3***	5	3*
		SS 11	SS 11	SS 7	SS 11	SS 7
Executive Functions						
Brixton	19 errors	8 errors	13 errors	25 errors***	6 errors	6 errors
	Moderate average	Superior	High average	Poor	Superior	Superior
Other						
HADS Anxiety (21)	9 mild anxiety	RSNA	0	2	3	9 mild anxiety
		Normal limits	Normal limits	Normal limits	Normal limits	
HADS Depression (21)	9 mild depression	RSNA	2	4	3	9 mild depression
		Normal limits	Normal limits	Normal limits	Normal limits	

Impaired test scores are shaded and severity of impairment is indicated with an asterisk(s): * = mild impairment, ** = moderate impairment, *** = marked impairment. Bracketed number is the maximum possible score for the given test based on version and number of trials completed. Standardized scaled scores (SS, mean = 10, SD = 3), neuropsychological *T* scores (TS, mean = 50, SD = 10), raw scores on Brixton (mean = 16, SD = 5.7), or percentiles (%iles) were provided from test normative data, matched for age and education, where available. RSNA = raw score not available, TNA = test not administered.

In terms of general cognitive functioning, all patients showed some degree of verbal and/or visuospatial memory impairment. Only patient P4 showed consistent, additional impairments on nonverbal reasoning, attention and executive function. With the exception of memory function, there was no cognitive domain that was consistently impaired across patients. To confirm this, we examined whether the patient group as a whole was significantly different from the norms within each domain, using Stouffer's method to calculate a combined *Z*‐statistic. The group was significantly impaired on both verbal (*Z* = −6.66, *P* < 2.8 × 10^−11^) and visuospatial memory (*Z* = −6.30, *P* < 3.1 × 10^−10^), but not verbal skills (*Z* = +1.17, *P* = 0.24), sustained attention (*Z* = −1.18, *P* = 0.24) nor executive function (Brixton, *Z* = +1.36, *P* = 0.17), and was actually above average on visuospatial skills (*Z* = +2.70, *P* = 0.0070).

### Grey‐Matter Analysis

The T1‐weighted structural scan for each patient was compared with their own set of age‐ and sex‐matched controls, where age was matched within 3–5 years of each patient's age.

#### ROI analysis of grey‐matter volume (GMV)

Table 3 shows the *T* values for comparison of patients versus controls (where negative *T* indicates smaller volume in patients) for the FreeSurfer subcortical ROIs, after collapsing left and right hemispheres and adjusting for total intracranial volume (for results split by hemisphere, see Supporting Information, Table 1; examples of the subcortical segmentation are shown in Supporting Information, Fig. 5). All six patients showed the predicted significant reduction in hippocampal volumes, with a mean volume that was 60% of that of controls. When correcting for multiple comparisons, three patients (P1, P4, and P6) also showed reduced amygdala volume, two showed reduced entorhinal volume (P4 and P6), one showed reduced parahippocampal volume (P6), and one showed reduced volume in pallidum (P2).

**Table 3:** Comparison of Each Patient (P1–P6) Against Their Own Group of Age‐ and Sex‐Matched Controls for the Volume of Subcortical Regions Estimated from T1‐Weighted Images Using FreeSurfer

Region	P1	P2	P3	P4	P5	P6
Control ages (and number)	51–61 (*n* = 41)	36–42 (*n* = 49)	63–69 (*n* = 45)	62–68 (*n* = 51)	52–62 (*n* = 41)	57–67 (*n* = 48)
Thalamus	−1.93 6.21/7.30 (85)	−0.17 7.83/7.93 (99)	−0.11 6.94/7.03 (99)	+0.82 7.68/7.19 (107)	+0.03 7.11/7.10 (100)	−1.62 6.72/7.61 (88)
Caudate	−0.51 3.46/3.71 (93)	−0.59 3.70/3.92 (93)	−0.53 3.17/3.52 (90)	+0.36 3.75/3.62 (104)	−1.26 2.98/3.34 (89)	+0.36 3.69/3.55 (104)
Putamen	−2.19 4.02/5.47 (74)*	−0.70 5.56/6.08 (92)	−1.21 4.42/5.07 (87)	−1.81 4.09/5.03 (81)	+0.46 4.92/4.73 (104)	−0.07 5.00/5.03 (99)
Pallidum	−1.39 1.20/1.48 (81)	−3.10 1.08/1.60 (67)**	−1.13 1.18/1.42 (84)	−1.29 1.20/1.43 (84)	+0.62 1.45/1.35 (107)	+1.26 1.66/1.44 (115)
Hippocampus	−5.07 1.97/4.28 (46)**	−4.81 2.53/4.44 (57)**	−3.32 2.31/3.99 (58)**	−1.80 3.19/4.06 (79)∼	−3.95 2.72/4.04 (67)**	−4.97 2.16/4.32 (50)**
Amygdala	−3.74 0.89/1.79 (50)**	−0.76 1.70/1.88 (91)	+0.10 1.67/1.65 (101)	−3.34 0.95/1.64 (58)**	−2.77 1.09/1.53 (71)*	−5.52 0.61/1.70 (36)**
Accumbens	−2.26 0.38/0.60 (63)*	−1.18 0.54/0.66 (82)	−0.30 0.48/0.51 (94)	−1.86 0.39/0.52 (65)	−2.40 0.32/0.48 (66)*	−0.36 0.45/0.48 (93)
Parahippocampal	−0.13 2.27/2.31 (98)	−2.52 1.75/2.51 (70)*	+1.07 2.42/2.15 (112)	−2.01 1.65/2.16 (76)	+0.18 2.11/2.07 (102)	−3.12 1.37/2.21 (62)**
Entorhinal	−1.76 0.96/1.22 (79)	−1.59 0.97/1.24 (79)	−0.85 1.03/1.17 (88)	−2.93 0.72/1.18 (61)**	−0.97 0.91/1.08 (85)	−3.67 0.62/1.18 (52)**

The first number in each cell is the *T* value, the middle two numbers are the mean volume in patient and controls, respectively (in units of 1,000 mm^3^), and the last number in brackets is percentage of mean control volume. ∼ indicates differences significant at *P* < 0.05 (one‐tailed for hippocampal predictions). * indicates differences significant at *P* < 0.05 (two‐tailed). ** indicates differences significant at *P* < 0.05 (two‐tailed) after Bonferroni correction for number (9) of ROIs tested. Shaded regions support prior predictions about Hippocampal volume loss, or survive correction for multiple comparisons.

We also examined all remaining FreeSurfer 30 cortical ROIs (averaged across hemisphere). Only one cortical ROI in one patient survived Bonferonni correction for the number of ROIs (i.e., 30) tested per patient: Pars Orbitalis in patient P6 (*T*(47) = −3.74, *P* = 5.0 × 10^−4^). Between 0 and 9 ROIs across patients survived *P* < 0.05 uncorrected, and therefore none was common across all patients. In the next section, we supplemented these ROI tests with an additional voxel‐wise analysis, using a different image‐based preprocessing stream (see Methods).

#### Voxel‐wise analysis

**Figure 1:**
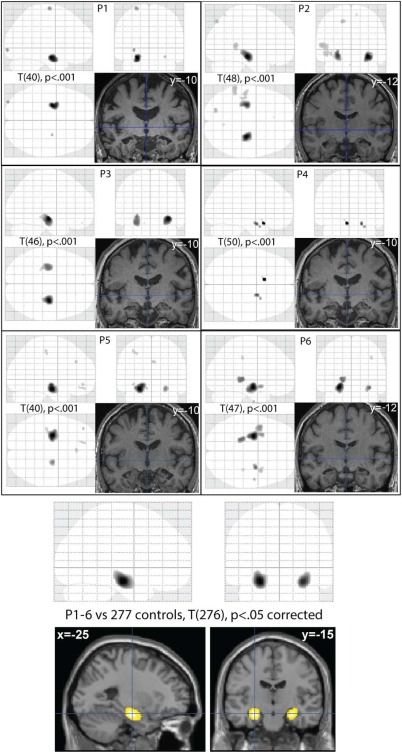
Top 6 panels show VBM results for each patient (P1–P6), *P* < 0.001 uncorrected for a T‐contrast testing for greater GMV in controls than patients (control groups individually matched to age and sex of patients, as in Table 1). Upper images in each panel are maximal intensity projections (MIPs) from three orthogonal views; bottom‐right image in each panel is a section through the normalized T1‐image of each patient (MNI *y*‐coordinate of coronal section given). Bottom panel shows group VBM results thresholded at *P* < 0.05 corrected, rendered on a T1‐weighted image of a canonical brain. [Color figure can be viewed at wileyonlinelibrary.com.]

The above ROI analyses confirmed that every patient had significantly reduced hippocampal volume, with no evidence for a consistent reduction in other subcortical or cortical ROIs. Nonetheless, to further check for grey‐matter reductions across the rest of the brain, we also performed a voxel‐wise analysis (VBM) on the same patient and control T1‐weighted images as in Table 3, after diffeomorphic registration and transformation to MNI space. The results for each patient separately are shown in the top panels of Figure 1. The only consistent region with GMV reduction was the anterior hippocampus (bordering on amygdala), with at least one of the left or right hippocampal clusters surviving small‐volume correction in all patients for the bilateral anatomical mask of the hippocampus. The bottom panel of Figure 1 shows the results of comparing the complete group of 277 controls with 6 patients (with each patient modeled separately). The bilateral hippocampal clusters, with peaks at (−26 −15 −18) and (+27 −14 −20), were the only regions to show significant reduction in GMV that survived whole‐brain correction. No areas showed GMV increases. These findings suggest that no region showed consistent volume changes across patients apart from the hippocampus.

### White‐Matter Analysis (Structural Connectivity)

Structural connectivity was assessed by DTI, comparing the group of 6 patients with a group of 36 controls (given that there were not enough DTI datasets using exactly the same MR sequence to construct control groups for each patient separately). The control group contained 17 males and ranged in age from 40 to 67. The mean age of controls (*M* = 55.3) did not differ significantly from that of patients (*M* = 57.8), *T*(40) = 0.59, *p* = 0.56. Given the imbalance in the patient group, gender was included as a covariate of no interest in all analyses.

#### ROI analysis of major hippocampal tracts

The FA values across the four WM ROIs revealed that only the Fornix showed significant reduction of about 25% in mean anisotropy in the patient group. Table 4 shows the results when using the “matched sample” WM skeleton template from the 6 patients and 6 randomly chosen controls (see Methods); identical patterns of significance were found when using the “full sample” template from all 42 scans (see Supporting Information, Table 2).

**Table 4:** Mean (and Standard Deviation) of FA and MD Values for WM ROIs, Together with Statistics from an Independent Sample *t*‐Test, with Gender as a Covariate of No Interest

WM ROI	Control mean (*N* = 36)	Patient mean (*N* = 6)	*T* (39)	*P*	% patient/control
FA					
Fornix (body)	0.346 (0.090)	0.257 (0.081)	−2.149	0.038*	75
Uncinate Fasc.	0.503 (0.044)	0.478 (0.048)	−1.345	0.186	94
Hipp. Cingulum	0.473 (0.040)	0.447 (0.045)	−1.018	0.315	96
Inf. Long. Fasc.	0.414 (0.024)	0.412 (0.019)	−0.049	0.961	100
MD (10^−3^ mm^2^ s^−1^)					
Fornix (body)	1.703 (0.401)	2.150 (0.610)	+2.357	0.024*	126
Uncinate Fasc.	0.719 (0.053)	0.758 (0.101)	+1.495	0.143	106
Hipp. Cingulum	0.674 (0.094)	0.748 (0.074)	+1.600	0.118	110
Inf. Long. Fasc.	0.741 (0.036)	0.775 (0.039)	+2.033	0.049*	105

These data used the “matched” skeleton from 6 patients and 6 controls; for results using “full” skeleton from all participants, see Supporting Information, Table 2. * = two‐tailed *P* < 0.05. Fasc. = Fasciculus; Hipp. = Hippocampus; Inf. = Inferior; Long. = Longitudinal.

The MD values also showed a difference in the Fornix, with an increase in mean diffusivity of about 25% in the patient group (Table 4). The inferior longitudinal fasciculus showed a smaller (approximately 5%), but also significant, increase in MD (which was almost significant when using the full sample template in Supporting Information, Table 2).

#### Voxel‐wise analysis (TBSS)

**Figure 2:**
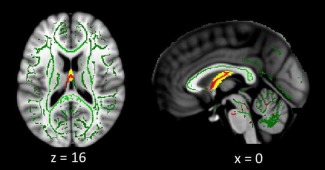
WM TBSS voxel‐wise results. Voxels showing a reduced FA in the patient group at *P* < 0.05 uncorrected are shown in red, superimposed on the matched WM skeleton in green and fornix body ROI in yellow. Voxels survived FDR correction for the fornix ROI, but no voxels survived correction for the other WM ROIs, nor across the whole skeleton. [Color figure can be viewed at wileyonlinelibrary.com.]

The above WM ROI analysis showed that there was a significant decrease in the average fornix FA across patients, but not in the other major white‐matter tracts connected to the hippocampus. To check for FA differences across the rest of the brain, we searched the whole WM skeleton using TBSS (again adjusting for gender). The results are shown in Figure 2. No voxels survived FDR correction elsewhere in the skeleton. The only voxels with reduced FA in the patient group that survived correction within our a priori WM ROIs were in the fornix ROI, with the peak at (0 −6 14, *T* = 3.28).

### Functional Connectivity

Functional connectivity was assessed by the linear dependency between fMRI timeseries from ROIs (see Methods), and comparing the group of 6 patients with a group of 44 controls. The control group contained 20 males and ranged in age from 36 to 70. The mean age of controls (*M* = 57.4) did not differ significantly from that of patients (*M* = 57.8), *T*(48) = 0.11, *P* = 0.91. Gender was again included as a covariate of no interest in all analyses.

One patient (P6), and two controls, were outliers in terms of movement (see Supporting Information, Fig. 1). Analyses were therefore repeated both with and without outliers, and differences noted below.

#### Functional ROI analysis of default mode network

Given that the hippocampus is known to be a key component of the DMN, we started by using functional ROIs within the DMN defined from prior resting‐state fMRI studies (Shirer et al., 2011): viz left and right hippocampus, left and right lateral parietal cortex, thalamus, midcingulate, posterior cingulate, and medial prefrontal cortex. This analysis revealed reduced connectivity between both left and right hippocampi and posterior cingulate (*T*(47) < −4.26, *P* < 0.001), which survived correction for the 28 pairwise comparisons. These functional connectivity reductions might be expected, given that reduced GMV in hippocampus is likely to reduce the signal‐to‐noise ratio in the hippocampal fMRI data, though note that the fMRI timeseries were based on a SVD that should minimize the effect of weak/noisy voxels (see Methods). There was also suggestion of reduced functional connectivity between DMN regions other than the hippocampi, such as between thalamus and (i) medial prefrontal, (ii) posterior cingulate, and (iii) left lateral parietal regions (*T*(47) < −2.21, *P* < 0.032), between posterior cingulate and (i) medial prefrontal and (ii) left inferior parietal cortex (*T*(47) < −2.36, *P* < 0.022), and between medial prefrontal and left parietal cortex (*T*(47) = −2.31, *P* < 0.025), though these would not survive correction for multiple comparisons. All the above comparisons also survived *P* < 0.05 when excluding the patient and two controls with excessive movement.

While these analyses suggest altered connectivity within regions previously associated structurally or functionally with the hippocampus, we next asked whether hippocampal lesions affected connectivity within and between the other, larger functional brain networks that many previous fMRI studies have identified.

#### Network‐based analysis

**Figure 3:**
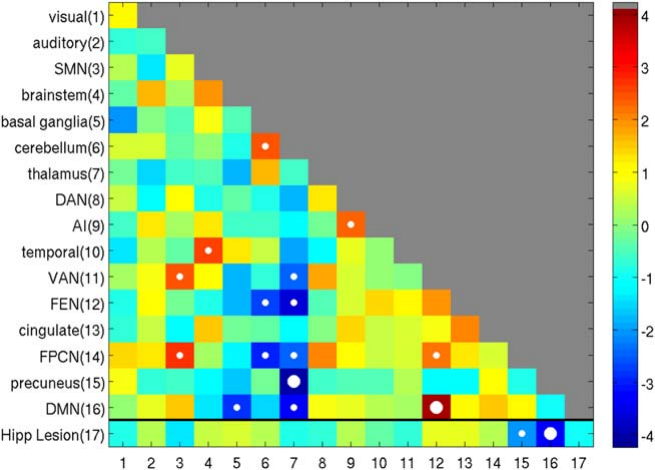
A network‐by‐network matrix of differences (*T*‐values) in functional connectivity between control and patient groups using the 16 canonical networks defined by Geerligs et al (2015), with addition of Hippocampal lesion site in current patients. Values on the leading diagonal reflect differences in within‐network connectivity; off‐diagonal values reflect differences in between‐network connectivity. Small white circles indicate significant differences between groups of *P* < 0.05, while large circles indicate those *P* values that survive Bonferroni correction. Color bar shows *T* values. SMN, somatomotor network; DMN, default mode network; DAN, dorsal attention network; AI, anterior insula network; VAN, ventral attention network; FEN, frontoexecutive network; FPCN, frontoparietal control network; Hipp, hippocampus. [Color figure can be viewed at wileyonlinelibrary.com.]

To see whether hippocampal lesions affect functional connectivity at the level of networks, we used the 16 “canonical” functional networks defined by Geerligs et al. (2015), which are based on fMRI data from a large number (*N* = 587) of population‐representative individuals acquired on the same scanner. In this network decomposition, the hippocampus falls within the “brainstem” network, while both the posterior cingulate seed of Hayes et al. (2012), and the lateral parietal region of Shirer et al. (2011), fall within the DMN. The *T*‐statistics for patients versus controls in their functional connectivity within and between each network are shown in Figure 3 (Supporting Information, Fig. 2 shows the matrices for each group separately).

The bottom row of Figure 3 shows differences between groups in their functional connectivity to the region of significant GMV hippocampal loss in patients (bottom panel of Fig. 1). Only one network showed a significant reduction in connectivity with the hippocampus that survived Bonferroni correction for 16 tests—the DMN—consistent with the general decreases found in the ROI analyses above.

**Figure 4:**
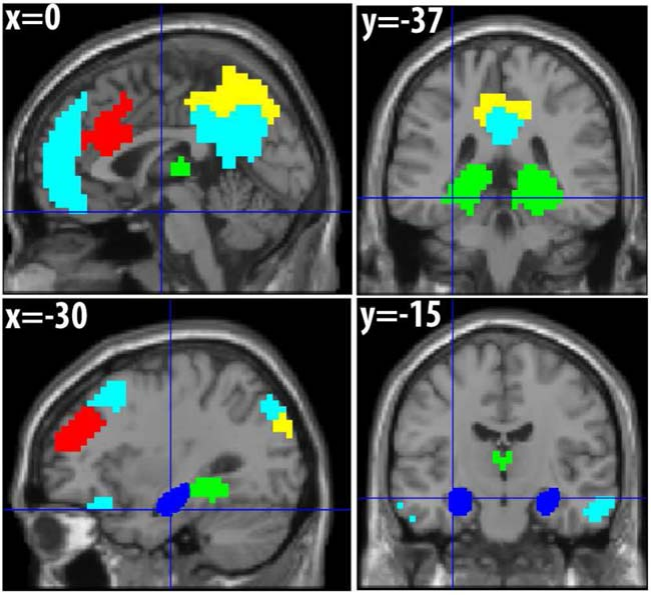
Functional networks. Light blue = default mode network (DMN); green = thalamic network; yellow = precuneus network; red = frontal executive network (FEN); dark blue = group hippocampal lesion (from Fig. 1). [Color figure can be viewed at wileyonlinelibrary.com.]

When examining the rest of the connectivity matrix in Figure 3, i.e., between and within the 16 resting‐state networks, two other between‐network connections also showed significant differences that survived correction for all 120 pairwise tests: (1) functional connectivity between the Thalamic network and Precuneus network was lower in patients, whereas (2) functional connectivity between the DMN and frontal executive network (FEN) was higher in patients. These networks are shown in Figure 4. Decreased functional connectivity with the thalamus is consistent with the strong connections between hippocampus and thalamus, and several other networks also show reduced functional connectivity with the thalamic network (but did not survive correction; see also Supporting Information, Fig. 2). The increased connectivity between DMN and FEN resembles the general increases in the seed‐based analyses of Hayes et al. (2012). Several other connections between, and within, cortical networks showed increased connectivity in the patients that did not survive correction, but are consistent with a general pattern of connectional diaschisis. Supporting Information, Figure 3 shows the same matrix with the three excessive movers excluded, which reproduced the same pattern of results that survived correction for multiple comparisons (in addition to increased connectivity within the cingulate network).

### Functional Connectomics

Finally, we explored the possibility of “connectomal diaschisis,” where the graph‐theoretic properties of a network change after a focal lesion. A simple measure of functional segregation is the difference in within‐network versus between‐network connectivity (Chan et al., 2014). For each participant, we therefore calculated the average of their leading diagonal elements minus the average of all off‐diagonal elements, for all 16 networks in Figure 3 (excluding the hippocampal lesion). When comparing these values across groups, we found significantly higher segregation in the patient group, *T*(48) = 2.03, *P* = 0.048 (the same was true when excluding the three excessive movers, *T*(45) = 2.28, *P* = 0.027). This was driven mostly by higher within‐network connectivity in the patients (*M* = 1.15) than controls (*M* = 1.05), rather than by differences in between‐network connectivity (*M* = 0.203 vs *M* = 0.215, respectively).

**Figure 5:**
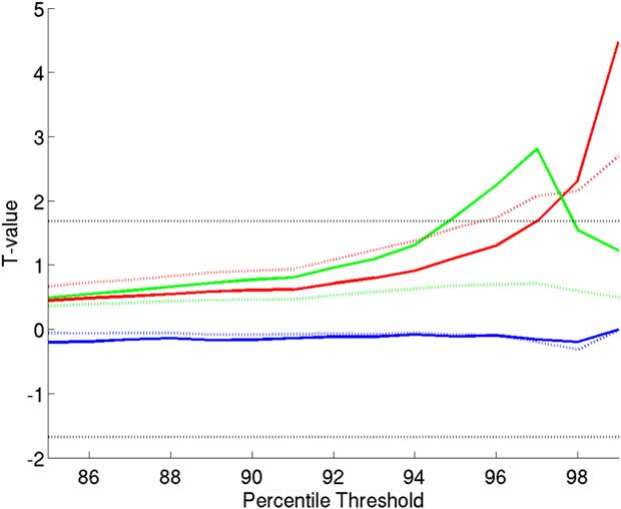
Graph‐theoretic measures from full ROI‐by‐ROI connectivity matrices. *T* values (solid lines) for patients minus controls on (1) clustering (red), (2) efficiency (blue), and (3) small‐worldness (green) as a function of connectivity percentile threshold (dotted black line shows *T* value for *P* < 0.05). Dotted colored lines show difference in mean values between groups (multiplied by 100 for visualization on same scale). [Color figure can be viewed at wileyonlinelibrary.com.]

Nonetheless, this measure assumes the same network definitions across participants. We therefore also calculated measures of clustering, efficiency and small‐worldness (see Methods), which all relate to the concept of functional segregation, on the original 716 × 716 ROI connectivity matrix for each participant. These measures operate on binarized connectivity matrices, to reflect the structure rather than strength of connections, so we explored a range of percentile thresholds for binarization from 85% to 99% (matching mean degree across individuals). The results are shown in Figure 5. The patients showed higher clustering and small‐worldness, at least for thresholds above 95%, which survived Bonferonni correction for 15 comparisons across thresholds in the case of clustering at the highest threshold (note this is a severe correction since tests are not independent, i.e., graph‐metrics are correlated across thresholds). The results were even more significant when excluding the three participants who moved excessively (see Supporting Information, Fig. 4), with differences in both clustering and small‐worldness surviving corrections for multiple comparisons, suggesting that the results do not reflect movement artifact. These results are consistent with the increased segregation found when using the canonical functional networks above.

## DISCUSSION

This study revealed clear effects of acquired hippocampal lesions on structural and functional connectivity across the human brain. Six individuals with amnesia from various etiologies were identified, in whom grey‐matter damage did not consistently extend beyond the hippocampus according to T1‐weighted MRI. Diffusion‐weighted and BOLD‐weighted MRI however showed differences in terms of (1) reduced structural integrity of the fornix, (2) decreases in “direct” functional connections with the lesion site, e.g., between hippocampus and posterior cingulate, (3) decreases in “indirect” functional connections between regions, and between networks, that do not include the hippocampus, e.g., between the thalamic and precuneus networks, (4) increases in indirect functional connections between the precuneus and frontal‐executive networks, and (5) increases in the functional segregation of the overall functional connectome. According to Carrera and Tononi's (2014) definitions, findings 3–4 correspond to connectional diaschisis and finding 5 corresponds to connectomic diaschisis. These findings extend previous research by virtue of identifying relatively focal hippocampal lesions (though see caveats below), and in examining whole‐brain functional connectivity and connectomics, beyond previous seed‐based or “hippocampal network” analyses.

### Grey‐Matter Loss

Though the most consistent evidence of GMV loss across our patients was in bilateral hippocampus, there are several reasons for caution. First, it is possible that our T1‐weighted MRI scan was unable to detect volume loss that existed in other brain regions. The specificity of hippocampal damage caused by limbic encephalitis—the case in three of our patients—is not clear, since abnormalities have been reported in other mediobasal temporal structures and insula (Herweh et al., 2007; Urbach et al., 2006). The other three patients suffered incidents with lack of oxygen, which is believed to have more selective effects on the CA1, CA2 and Dentate Gyrus of the hippocampus, with little evidence of other damage in the MTL following ischemic lesions in monkeys (Zola‐Morgan et al., 1992). Human developmental amnesia following hypoxia, on the other hand, has been associated with MRI volumetric reductions not only in hippocampus, but also in posterior thalamus, putamen, and retrosplenial cortex (Vargha‐Khadem et al., 2003). Using similar methods, our adult‐onset cases had comparable volume loss in hippocampus to developmental cases (40%), but did not show any evidence for extrahippocampal damage that was consistent across our six patients. One possibility is that anoxia during early years of life (at least to age 14) leads to more extensive damage (see also Meng et al., 2014).

Second, our more focused MRI volumetric analysis of subcortical regions did reveal significant loss outside the hippocampus in some of our cases. For example, the amygdala showed GMV reduction in 4 of our patients (3 of which survived correction for multiple comparisons). The border between amygdala and anterior hippocampus can be difficult to detect with MRI, and it is possible that this amygdala change was a consequence of shrinkage in nearby parts of the hippocampus, rather than cell loss in the amygdala per se. The same registration problem potentially applies to the entorhinal and parahippocampal volumes that were reduced in 2–3 of our cases, though of course it is also possible that there was true cell loss in all three of these structures. Thus, we do not wish to claim that the connectivity differences we observed must be due solely to hippocampal damage; our claim is only that the hippocampus is the most likely cause, because it is the brain region that showed the most consistent volume loss across our six cases. Indeed, it is likely that initial damage to the hippocampus causes grey‐matter loss in other connected regions, owing to the impoverished structural and/or functional connectivity that ensues.

### White‐Matter Loss

When using FA, as the most common measure of WMI, a significant reduction in patients was only found in the fornix; not in the other major WM tracts related to the hippocampus (i.e., hippocampal cingulum, inferior longitudinal fasciculus and uncinate fasciculus). This apparently selective WM damage is consistent with previous nonhuman (Meng et al., 2014; Shamy et al., 2010) and human (e.g., Liao et al., 2011; Voets et al., 2009) DTI studies. In a previous study of acquired human hippocampal lesions, significant fornix damage was not found in the one case with a focal hippocampal lesion (only in the case with more extensive MTL lesions; Rudebeck et al., 2013). It is possible that the greater statistical power obtained from combining 6 cases as a group was necessary to detect fornix changes in this study. Like Rudebeck et al. (2013) though, we did not find FA changes elsewhere using voxelwise analysis (TBSS).

In terms of other white‐matter tracts, Shamy et al. (2010) acquired DTI data 2 years after a neurotoxic lesion in adult macaques, which reduced hippocampal volume to 72% of controls, but showed no evidence of damage to nearby regions. Abnormal diffusion metrics were only found in the fornix and ventromedial prefrontal cortex, with no detectable effect on other pathways like the uncinate fascicle. They argued that the lack of effect on uncinate fascicle is expected because it carries mainly reciprocal projections between inferior temporal cortex and lateral and orbital prefrontal cortex (Ungerleider et al., 1989), lacking a major hippocampal contribution, and the same might apply to our study with humans.

Meng et al. (2014) found more extensive white‐matter changes following similar lesions in NHP, not only in the fornix, but also the temporal stem (including uncinate fasciculus) and optic radiations. They speculated that this additional WM damage may reflect the fact that their lesions were performed in neonates (to compare to human cases of developmental amnesia), resulting in abnormal maturation or functional activity/connectivity post lesion. This may explain why we did not see changes in the uncinate fasciculus, nor hippocampal cingulum or inferior longitudinal fasciculus, after adult‐onset lesions.

However, while FA is one of the most common measures used in DTI, we should note that it is not the only, or necessarily the best, measure of WMI (Jones et al., 2013), and other white matter tracts could be affected by hippocampal lesions in ways that we were unable to detect. Indeed, when using a complementary measure of mean diffusivity (MD), instead of FA, the inferior longitudinal fasciculus did show evidence of a small but significant increase in the patient group. Given that the same ROI did not show a significant difference in FA (in the same way the fornix did), and given the number of comparisons made on the DTI metrics, we refrain from interpreting this MD difference in the inferior longitudinal fasciculus, and await future replication studies. More importantly, rather than claiming there is no evidence for loss in structural connectivity outside the fornix, the more interesting findings from this study concern the significant evidence that was found for changes in functional connectivity.

### Connectional Diaschisis

Our functional connectivity results replicated a number of previous studies in terms of reduced connectivity between the (damaged) hippocampus and other “directly” connected regions like the posterior cingulate (Liao et al., 2011; Voets et al., 2009; Rudebeck et al., 2013; Hayes et al., 2012). This is consistent with the above fornix damage, since many of the connections between the hippocampus and cortical and subcortical regions are conveyed via the fornix (Aggleton and Saunders, 1997). Alternatively, the reduced functional connectivity could simply reflect reduced signal or higher noise in the functional activity of the hippocampus, resulting in weaker statistical dependency with activity in other regions.

We also found a suggestion of reduced functional connectivity between other ROIs within the DMN, such as between thalamus and posterior cingulate, and between posterior cingulate and inferior parietal cortex, which is less easy to explain simply by reduced signal‐to‐noise ratio of activity in these regions (given lack of evidence for GMV loss in these regions). This “remote diaschisis” between regions is more likely to reflect disruption of the whole hippocampal/default‐mode network, for example owing to fornix damage. Moreover, the network analysis showed that this disruption was found not only within a functional network, but also across functional networks, specifically between the thalamic and precuneus networks.

Further still, this “disruption” caused increased, as well as decreased, connectivity between some networks, suggesting more complex effects of hippocampal dysfunction than simply weakened structural connections (e.g., some form of functional “disinhibition”). Unlike Hayes et al. (2012), we did not find evidence of any increased connectivity within the DMN, but we did find increased connectivity between the DMN as a whole and an FEN. This pattern suggests that hippocampal lesions can have extensive “knock‐on” effects on communication between hippocampal networks and frontal networks.

### Connectomic Diaschisis

Finally, when examining functional segregation of the canonical whole‐brain networks, defined as the difference in within‐ versus between‐network connectivity (Chan et al., 2014), we found evidence of more segregated networks in the patient group. Moreover, when examining graph‐theoretic measures of the complete region‐by‐region connectivity matrix, we found increased clustering and small‐worldness in the patient group, at least with high connection thresholding (i.e., relatively sparse graphs). These increases in segregation are what simulations based on NHP structural connectivity predict, following deletion of major “connector hubs” (brain regions that are highly connected to regions in several other networks; Sporns et al., 2007). This result is also consistent with a prior finding that only lesions that affect such connector hubs (mainly fronto‐parietal, following stroke) lead to increased modularity (Gratton et al., 2012).

### Implications

Analyses of structural connectivity have not typically revealed the hippocampus as a member of the “rich club” of highly connected hubs (e.g., van den Heuvel et al., 2012; though see Kocher et al., 2015). Nonetheless, simulations of functional connectivity have shown that it can act like a hub, owing to the convergence of many cortical inputs (Misic et al., 2014). This central role can explain why hippocampal lesions affect functional connectivity as extensively as observed in the present study, despite the minimal detectable loss of structural connectivity. The loss of general brain‐wide functional integration that follows (i.e., increased functional segregation) may contribute to the amnesia observed in our patients, though it is noteworthy that lesions within the DMN, such as the hippocampus, were not associated with extensive cognitive impairment when pooled across patients with a diverse range of brain injuries (Warren et al., 2014). Rather, the latter study implicated regions in the prefrontal cortex and insula with impairments across a broad range of cognitive domains. Lesions to the hippocampus may produce a more selective impairment in one domain, at least in the patients here, namely memory.

In addition to the reduced functional connectivity to/from the hippocampus, a likely cause of the memory impairments in the present patients is the reduced functional connectivity between the Thalamus and Precuneus networks. These networks involve regions that have been associated with memory circuits (see Jeong et al., 2015, for review). The behavioral significance of the increased connectivity between DMN and FEN, on the other hand, is less clear. While an inverse relationship has been noted between the DMN and frontal‐parietal networks (Fox et al., 2005), and this relationship linked to behavioral variability in response times (Kelly et al., 2008), the frontal‐parietal regions associated with this “competitive” interaction with DMN correspond more closely to our DAN than our FEN. Moreover, our FEN did not show a negative (anticorrelated) relationship with DMN within either the controls or patient groups. The cognitive implications of the increased DMN–FEN connectivity in this study therefore deserve further investigation.

### Limitations

As with any empirical study, the present data have limitations. Foremost, we were only able to test six patients with acquired lesions that appeared to be confined mainly to hippocampus. This reflects the relative rarity of patients with such specific damage. Nonetheless, as far as we are aware, our sample size is greater than other MRI studies of this type of patient.

Second, DWI measures of WMI are indirect and imperfect. For example, free‐water can contaminate delineation of fibers near to ventricles (CSF) such as the fornix and cingulum. Indeed, more sophisticated analysis methods (e.g., Pasternak et al., 2009) and improved multi‐shell DWI sequences (e.g., Hoy et al., 2014) that minimize free‐water contamination may increase delineation accuracy of the fornix in future studies.

Third, we have only explored connectional and connectomic diachsisis in terms of functional connectivity. Similar methods could be applied to tractographic analysis of our DTI data, to estimate the structural connectome throughout the brain (rather than just the major fiber bundles examined here). We deliberately refrained from DTI tractography in this study, because of the difficulties with this technique, and nonoptimality of our single‐shell DTI data (Sotiropoulos et al., 2013). Fourth, functional connectivity as measured by fMRI, or more precisely, the linear statistical dependency metric used here (*Z*‐statistic), is an indirect measure of neural connectivity (e.g., may be altered by vascular changes in patients, for example owing to their medication) and does not capture more complex (e.g., nonlinear) dependencies. Furthermore, because this connectivity was measured at rest, as common in fMRI studies, it is also possible that the effects we found on functional connectivity in our patients reflect state‐dependent differences. It would be interesting to see therefore whether the present effects of hippocampal lesions remain when measured in other states (see e.g., Geerligs et al., 2015); a form of “functional diaschisis” (Price et al., 2001).

We have also not explored distinctions between left and right hippocampus (since our patients tended to have bilateral damage), nor between anterior and posterior hippocampus (Strange et al., 2014), which may show different patterns of connectivity (Libby et al., 2012). The VBM analysis suggested that the GMV loss was more significant (across patients) in anterior hippocampus, though this should be treated with caution because of partial volume effects (the hippocampus is “thinner” toward its caudal end, and VBM analyses require a minimal amount of spatial smoothing in order for valid statistical inference, which may have diluted statistical significance of GMV loss in more posterior regions). Future studies could explore such left/right and anterior/posterior distinctions further.

Finally, we are unable to tell whether the connectional/connectomal diaschisis reported here relates to clinical outcome, i.e., whether it reflects a pathological state or compensatory mechanism (Carrera and Tononi, 2014). All the present MRI data were acquired several years after the lesion, suggesting that they are unlikely to reflect short‐term compensation/recovery processes. Standard neuropsychological tests showed that memory was the only cognitive domain consistently impaired across the group, though the small number of individuals meant there was little point in examining correlations across patients between their brain measures and their neuropsychological scores, and it is possible that more subtle cognitive deficits may be revealed by other tests. Indeed, we plan to examine the behavioral/cognitive profiles of these patients in more detail in future studies, and to relate these profiles to the neural findings reported here. Nonetheless, the present data clearly demonstrate the extensive “knock‐on” effects of hippocampal lesions, which is important knowledge for neuropsychological, epilepsy, and dementia communities.

## Supporting information

Supporting Information
