# Supplementary material for: The effects of hippocampal lesions on MRI measures of structural and functional connectivity
Source: Hippocampus. 2016 Aug 24;26(11):1447–63. doi: 10.1002/hipo.22621 (PMC5082505; doi:10.1002/hipo.22621)

# Supplementary Material

### Supplementary Table 1

GMV subcortical ROI analyses, now split by Hemisphere. T-value for comparison of each patient (P1-P6) against their own group of age- and sex-matched controls for the volume of sub-cortical regions estimated from T1-weighted images using FreeSurfer. Figure in brackets is percentage of mean control volume. * indicates differences significant at p<.05 (two-tailed). ** indicates differences significant at p<.05 (two-tailed) after Bonferroni correction for number (18) of ROIs tested. Shaded regions support prior predictions about Hippocampal volume loss, or survive correction for multiple comparisons. Parahip = Parahippocampus; EnthC = Entorhinal Cortex.

| Region | **P1** | **P2** | **P3** | **P4** | **P5** | **P6** |
| --- | --- | --- | --- | --- | --- | --- |
| Control ages  (and number) | 51-61  (n=41) | 36-42  (n=49) | 63-69  (n=45) | 62-68  (n=51) | 52-62  (n=41) | 57-67  (n=48) |
| Left Thalamus | -1.56  (85) | -1.11  (90) | +0.01  (100) | +1.01  (110) | -0.21  (98) | -1.85  (83) |
| Right Thalamus | -1.92  (85) | +1.14  (108) | -0.26  (97) | +0.37  (103) | +0.32  (102) | -0.84  (94) |
| Left Caudate | -0.24  (97) | -0.71  (92) | -0.41  (93) | +0.38  (104) | -1.27  (89) | +0.38  (104) |
| Right Caudate | -0.72  (90) | -0.47  (94) | -0.61  (88) | +0.29  (103) | -1.16  (89) | +0.32  (103) |
| Left Putamen | -2.02  (73)* | -0.62  (93) | -1.12  (88) | -2.19  (78)* | +0.66  (107) | -0.08  (99) |
| Right Putamen | -2.20  (74)* | -0.73  (90) | -1.13  (87) | -1.18  (84) | +0.11  (101) | -0.06  (99) |
| Left Pallidum | -0.88  (83) | -1.71  (76) | -0.42  (92) | -1.74  (73) | +0.29  (105) | +1.11  (119) |
| Right Pallidum | -1.88  (79)+ | -3.75  (59)** | -1.79  (76) | -0.43  (94) | +1.12  (109) | +1.02  (112) |
| Left Hippocampus | -5.57  (43)** | -4.27  (58)** | -2.69  (60)* | -1.13  (84) | -3.87  (63)** | -6.92  (28)** |
| Right Hippocampus | -4.03  (49)** | -4.84  (57)** | -3.21  (56)** | -2.09  (73)* | -3.67  (71)** | -2.62  (72)* |
| Left Amygdala | -3.49  (45)** | -1.32  (84) | +0.40  (105) | -3.05  (60)* | -3.33  (59)** | -5.20  (36)** |
| Right Amygdala | -3.19  (55)* | -0.22  (97) | -0.17  (97) | -2.72  (56)* | -1.64  (83) | -4.46  (35)** |
| Left Accumbens | -1.80  (64) | -1.11  (80) | -0.17  (95) | -1.73  (58) | -3.78  (38)** | -0.19  (95) |
| Right Accumbens | -2.37  (62)* | -1.05  (84) | -0.40  (92) | -1.53  (72) | -0.73  (89) | -0.49  (91) |
| Left Parahip | +0.15  (102) | -2.09  (73)* | +0.25  (104) | -1.29  (78) | -0.17  (98) | -3.74  (56)** |
| Right Parahip | -0.38  (94) | -2.36  (66)* | +1.59  (122) | -1.92  (75) | +0.55  (106) | -1.97  (69) |
| Left EnthC | -1.48  (82) | -1.29  (80) | +0.04  (101) | -2.00  (73) | -0.62  (89) | -5.35  (27)** |
| Right EnthC | -1.50  (76) | -1.35  (77) | -1.28  (75) | -2.71  (48)* | -1.19  (80) | -1.08  (82) |

### Supplementary Table 2

Mean (and standard deviation) of FA values for White Matter (WM) ROIs, together with statistics from an independent sample t-test. These data used the “full” skeleton from 6 patients and all 44 controls; for results using “matched” skeleton from 6 controls, see Table 4. * = two-tailed p<.05. ~ =one-tailed p<.05. Fasc. = Fasciculus; Hipp. = Hippocampus; Inf. = Inferior; Long. = Longitudinal.

### Supplementary Figure 1


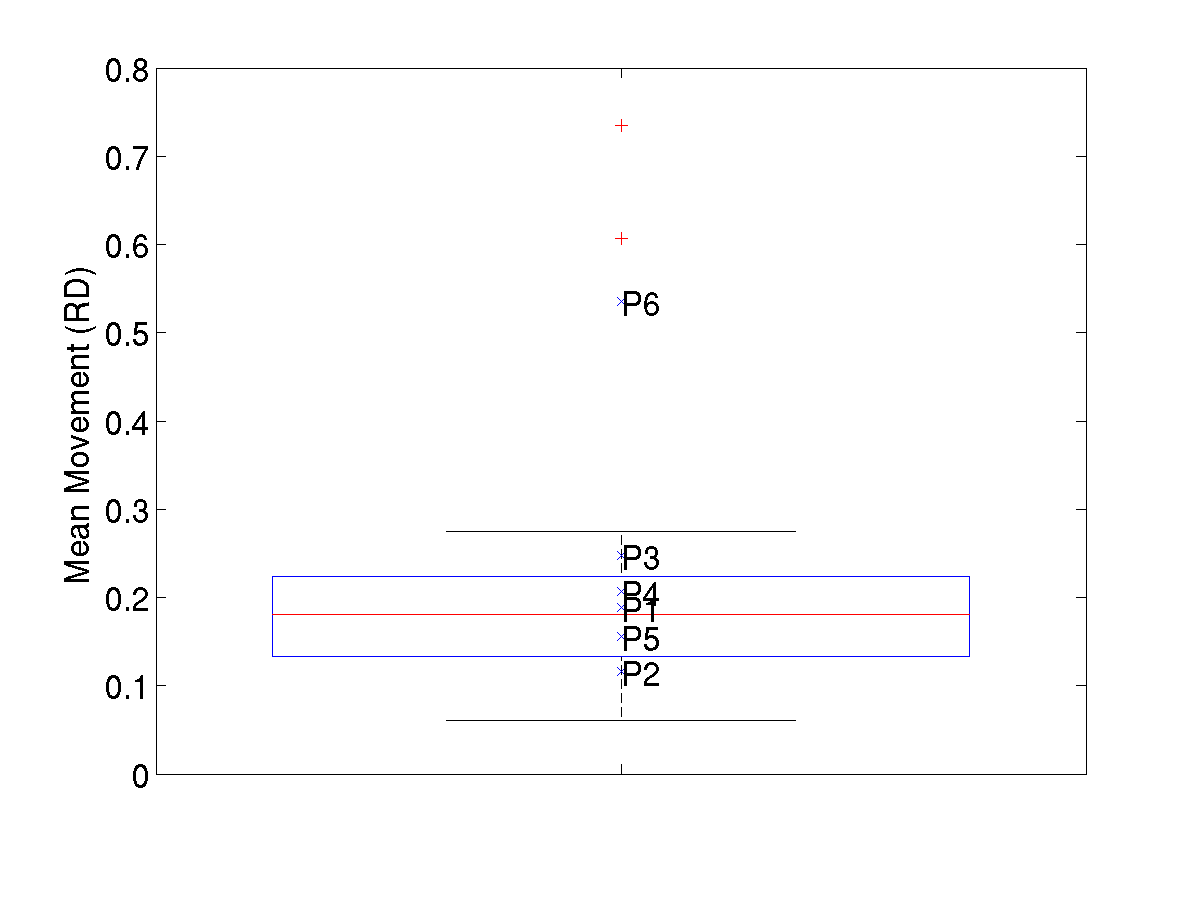


Supp Figure 1. Boxplots for mean movement (RD) across the resting-state fMRI session. Red line shows median, the blue box encompasses the 25% and 75% percentiles, the black bars (whiskers) represent extreme values and the (two) red crosses are deemed outliers for the N=44 controls. The patients are numbered individually, one of which (P6) is also an outlier.

### Supplementary Figure 2


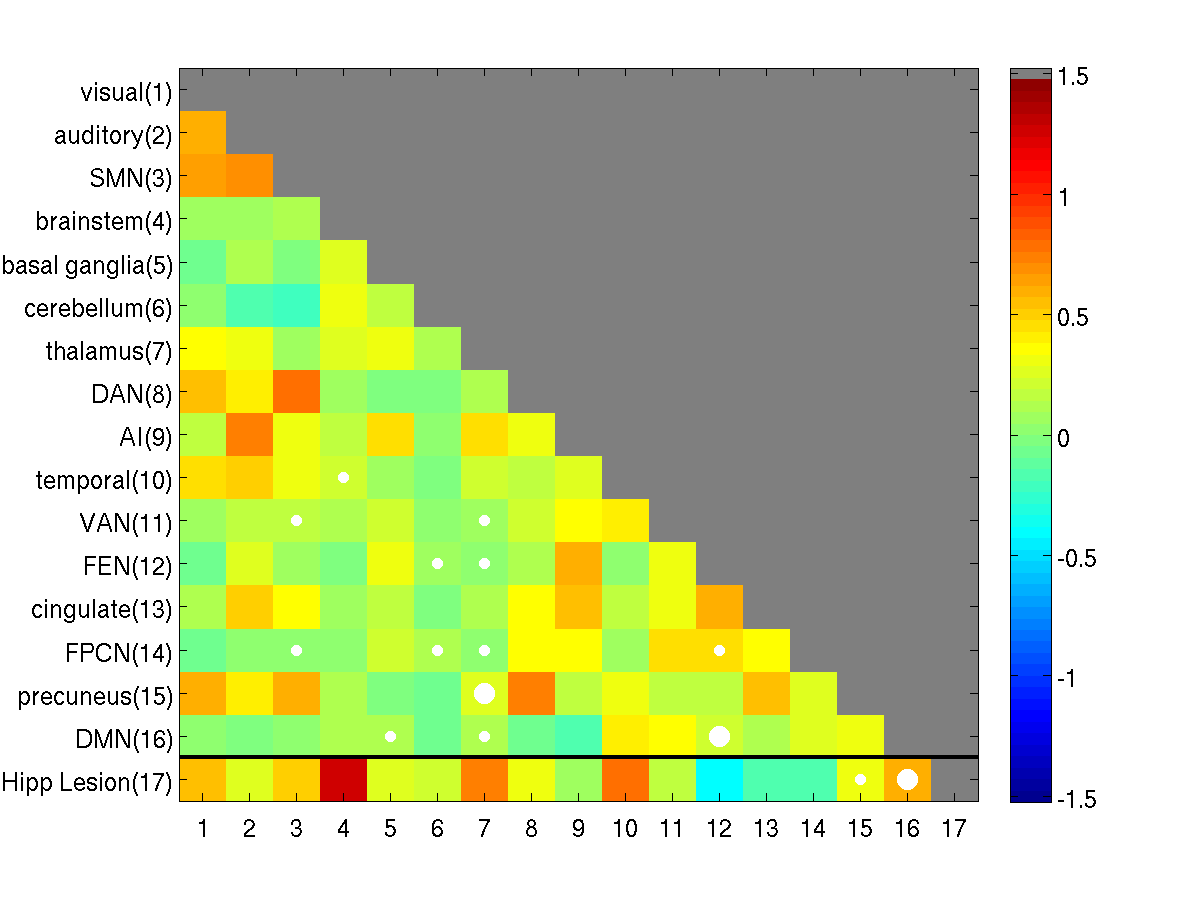


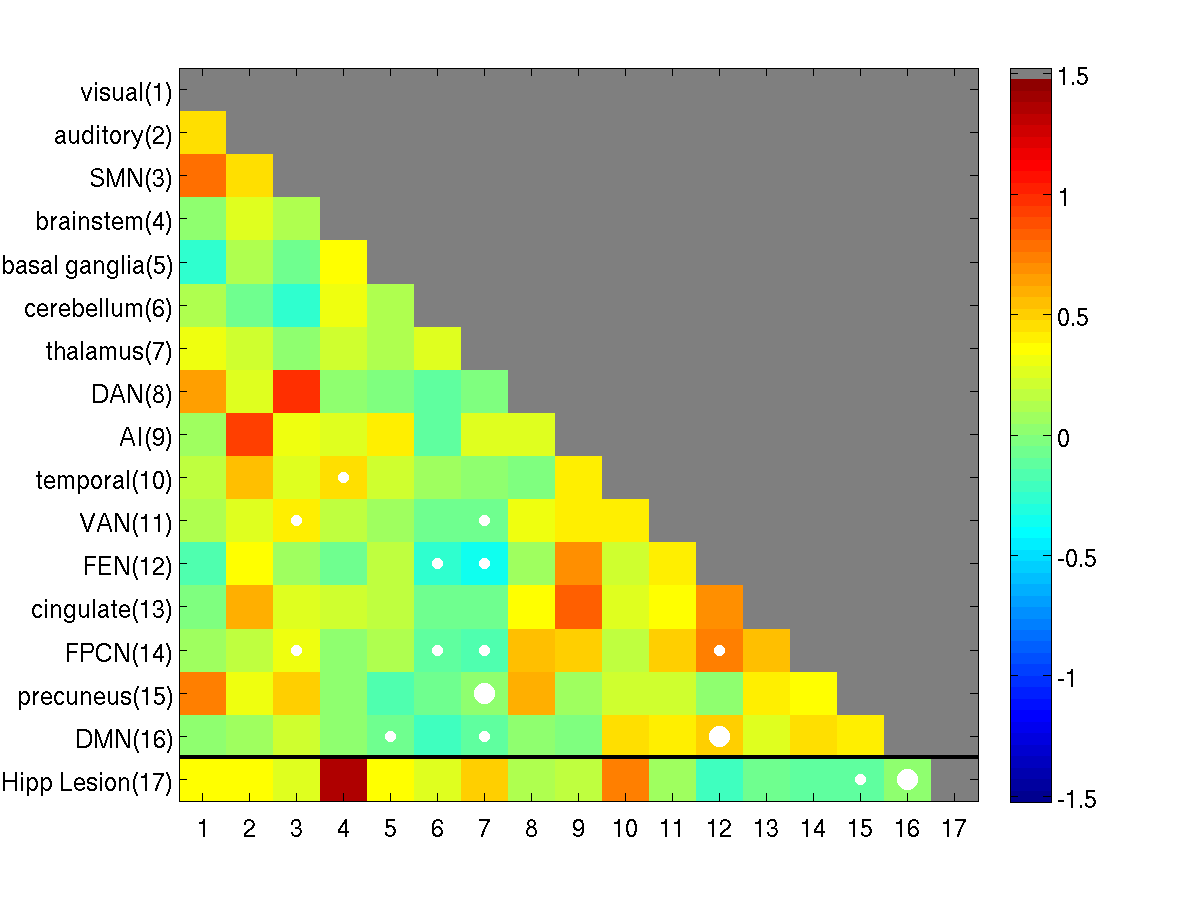


Supp Figure 2. A network-by-network connectivity matrix of mean connectivity for each group separately (from which T-values for group differences in Figure 3 were generated) for all 44 controls (top) and all 6 patients (bottom). Colourbar shows Z-statistics for mean connection “strengths”. Within-network connectivity (leading diagonal) not shown, since uniformly high by definition. White circles represent significant differences between groups from Figure 3 legend for more details.

### Supplementary Figure 3


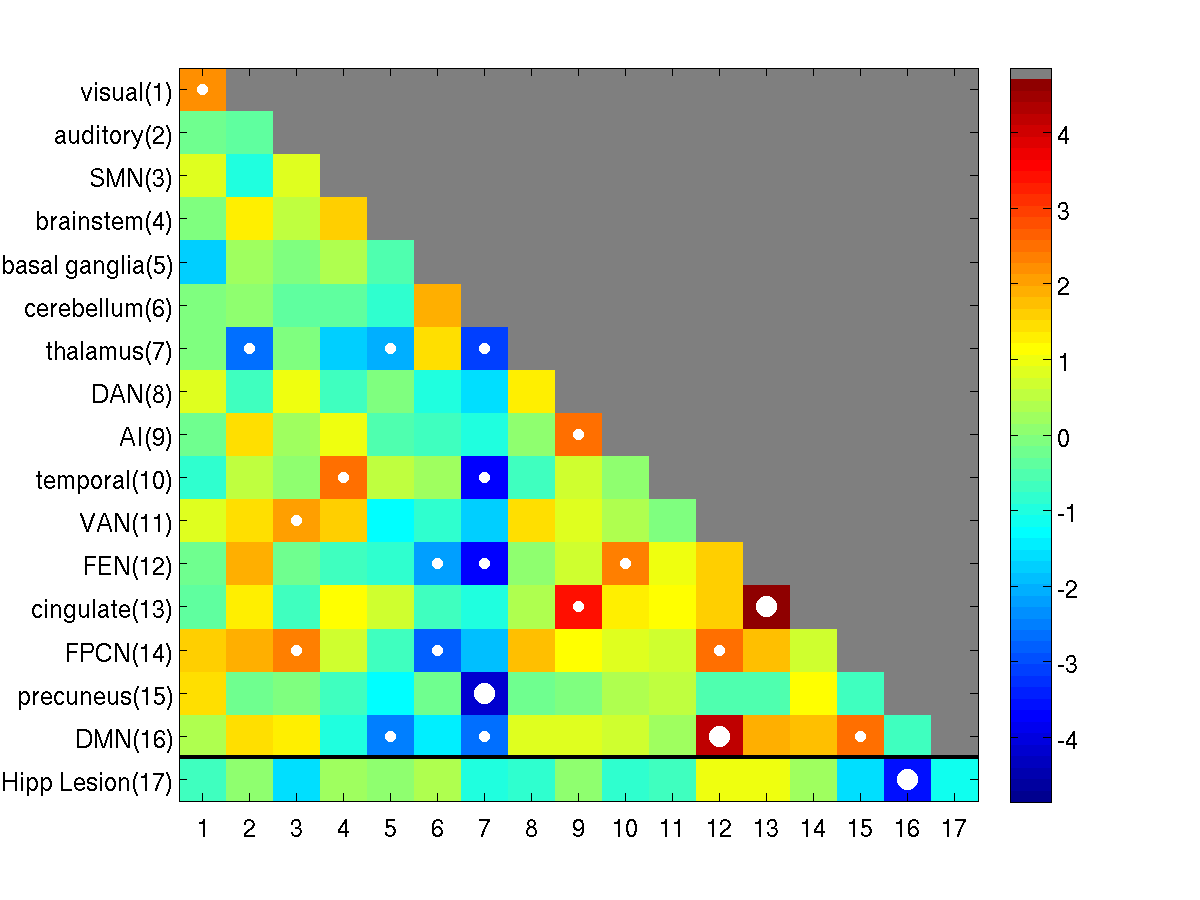


Supp Figure 3. A network-by-network connectivity Matrix excluding the one patient and two controls who moved excessively in Supp Figure 1. Colourbar shows T-values. Results that survive Bonferroni correction are the same as when the patient and two controls are included (Figure 3), except now additional increases in functional connectivity in patients within the cingulate network.

### Supplementary Figure 4


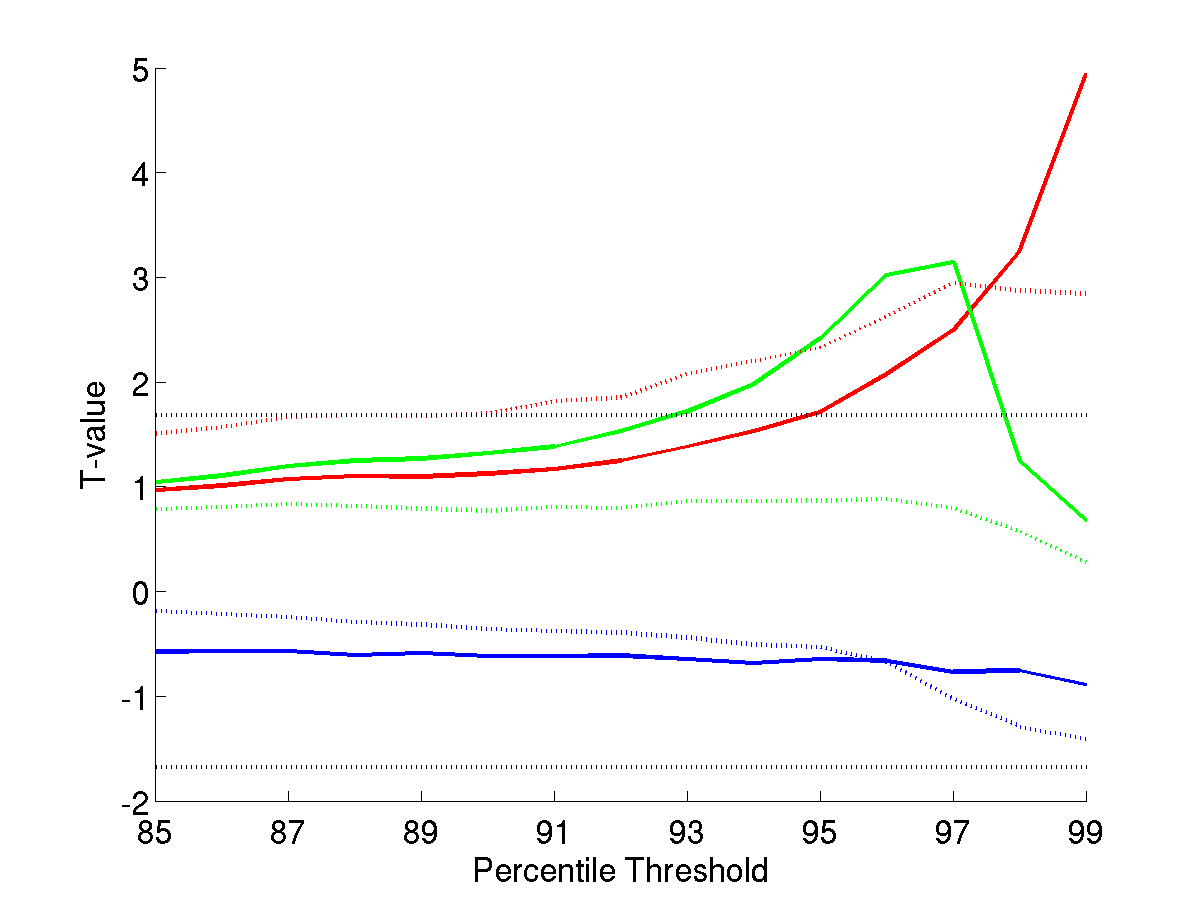


Supp Fig 4. Graph-theoretic measures from full ROI-by-ROI connectivity matrices. T-values (solid lines) for patients minus controls on 1) clustering (red), 2) efficiency (blue) and 3) small-worldness (green) as a function of connectivity percentile threshold (dotted black line shows T-value for p<.05), after removing the one patient and two controls who moved excessively in Supp Figure 1. Dotted coloured lines show difference in mean values between groups (multiplied by 100 for visualisation on same scale).

### Supplementary Figure 5: Example of subcortical segmentations for example patient. Images were created using the FreeSurfer tool “Tkmedit” (https://surfer.nmr.mgh.harvard.edu/fswiki/FsTutorial/Tools).

**P1** Red cross hair: Talairach coordinate [-23, -10, -18]


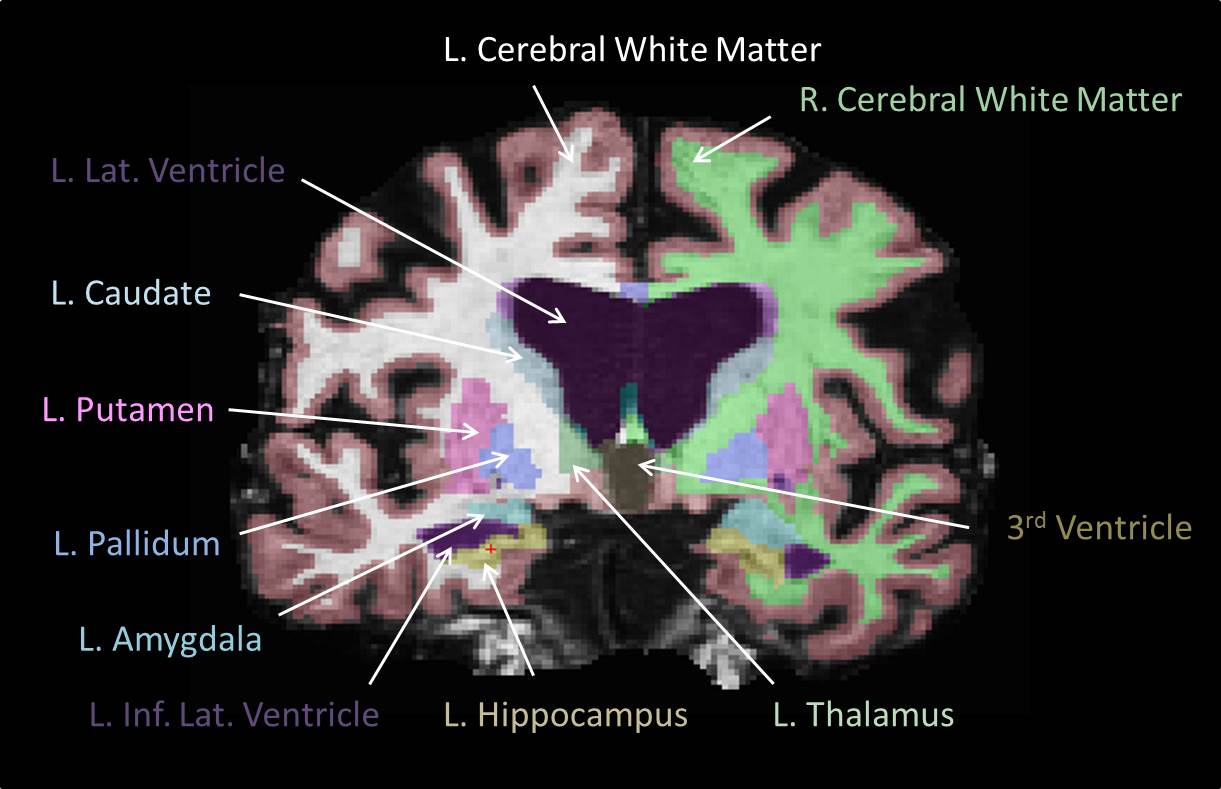


**P2** Red cross hair: Talairach coordinate [-23, -10, -18]


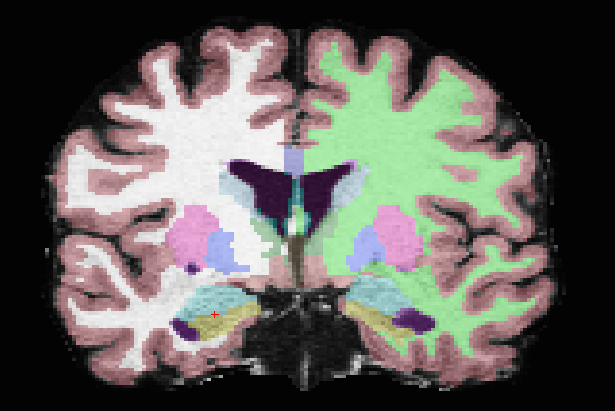


**P3** Red cross hair: Talairach coordinate [-23, -16, -16]


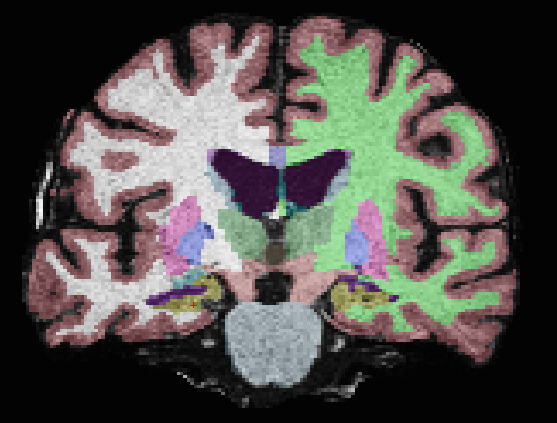


**P4** Red cross hair: Talairach coordinate [-23, -12, -18]


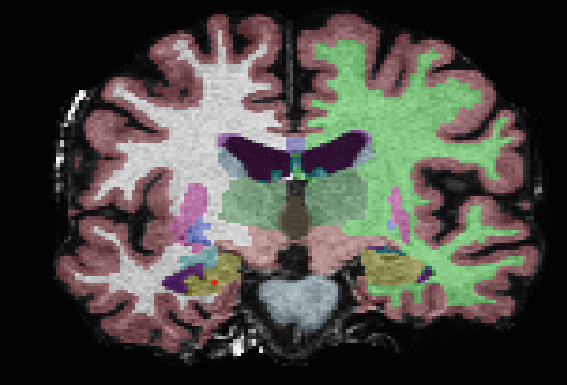


**P5** Red cross hair: Talairach coordinate [-23, -13, -18]


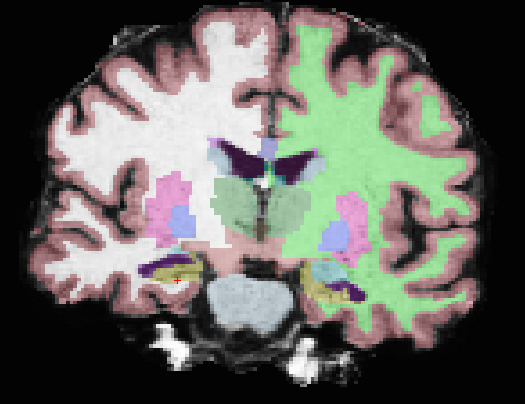


**P6** Red cross hair: Talairach coordinate [-23, -13, -18]


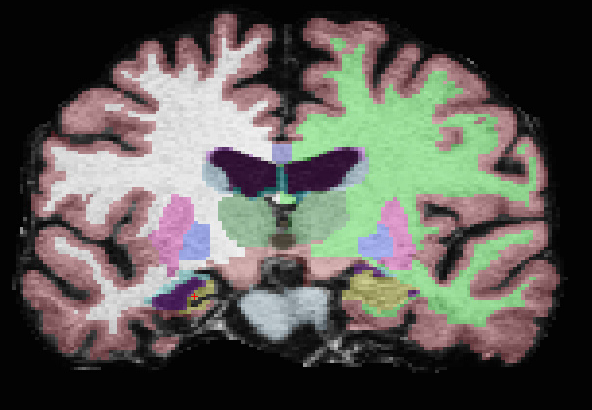

Supplement: Supplementary file 1 — Supporting Information [file HIPO-26-1447-s001.docx]
